# Recombinant E2 protein enhances protective efficacy of inactivated bovine viral diarrhea virus 2 vaccine in a goat model

**DOI:** 10.1186/s12917-018-1520-2

**Published:** 2018-06-19

**Authors:** Yao-Chi Chung, Li-Ting Cheng, Jia-Yu Zhang, Yue-Jyun Wu, Shyh-Shyan Liu, Chun-Yen Chu

**Affiliations:** 1 Graduate Institute of Animal Vaccine Technology, College of Veterinary Medicine, National Pingtung University of Science and Technology, 1, Shuehfu Road, Neipu, Pingtung 91201 Taiwan; 2 Department of Veterinary Medicine, College of Veterinary Medicine, National Pingtung University of Science and Technology, 1, Shuehfu Road, Neipu, Pingtung 91201 Taiwan

**Keywords:** BVDV, Inactivated vaccine, Subunit vaccine, E2

## Abstract

**Background:**

Inactivated and subunit bovine viral diarrhea virus (BVDV) vaccines have shown limited protective efficacy. This study aimed to evaluate the effectiveness of a vaccine containing both inactivated BVDV (iBVDV) and baculovirus-expressed recombinant E2 (rE2), an important BVDV antigen with strongly neutralizing epitopes.

**Results:**

Four groups of goats were immunized twice with one of four vaccine preparations: 1) iBVDV+rE2, 2) rE2, 3) iBVDV, and 4) saline, and challenged with BVDV. For goats vaccinated with the iBVDV+rE2 vaccine, no viremia was observed after challenge, and clinical signs, pyrexia, and leukopenia were reduced compared to the saline group. In contrast, for goats vaccinated with either iBVDV or rE2 alone, viremia was still detectable.

**Conclusion:**

The combination of iBVDV and rE2 elicited stronger protective immune responses against BVDV than iBVDV or rE2 alone.

## Background

Bovine viral diarrhea virus (BVDV) infects immune cells and causes short-term leukopenia, immunosuppression, pyrexia, and diarrhea in infected animals [1]. It is strongly associated with the bovine respiratory disease complex that can result in severe economic losses for the cattle industry [2]. Infection of pregnant cattle with BVDV can result in abortions and stillbirths. BVDV belongs to the *Pestivirus* genus of the *Flaviviridae* family and its single-stranded positive-sense RNA genome encodes one polyprotein that is cleaved into 11 or 12 viral proteins, including envelope proteins (E^rns^, E1, and E2), the capsid protein (C), and nonstructural proteins. Based on the nucleotide sequences of the 5′ untranslated region, BVDV can be classified into two species, BVDV-1 and BVDV-2, each species containing a number of subgenotypes. The high genetic diversity of BVDV makes controlling of disease difficult [3]. In Taiwan, only BVDV-2 has been reported.

Both inactivated and subunit vaccines have been developed against BVDV, but neither offers complete protection. With inactivated BVDV (iBVDV) vaccines, protection comes mainly from humoral response directed at the E2 protein, and the duration and range (across different serotypes) of protection are limited [3]. When the E2 protein is used as a subunit vaccine, only partial protection is observed (lack of pyrexia and reduction in both leukopenia and nasal virus shedding) [4, 5].

In this study, we evaluated the protective efficacy of a vaccine that included both iBVDV and baculovirus-expressed, recombinant E2 (rE2) protein. Since data indicated that the E2 protein serves as the major antigen with neutralizing epitopes [3], we hypothesized that the effectiveness of iBVDV vaccines, which have production titers limited at around 10^8^ FAID_50_/mL (50% fluorescent antibody infectious dose), can be enhanced by the addition of rE2. Furthermore, baculovirus expression of E2 protein in insect cells allows for post-translational modifications, such as protein folding and glycosylation. A water-in-oil-in-water (w/o/w) adjuvant was used for prolonged antigen presentation [6]. Immunization and challenge experiments were performed on goats since they are smaller and more economical for the evaluation of various BVDV vaccine formulations.

## Methods

### BVDV strains and virus titer determination

A BVDV-2 strain, BVDV/TW 2008, was obtained from the Animal Health Research Center, Council of Agriculture, Taiwan, and cultured for E2 gene cloning. For iBVDV vaccine production and to be used as the challenge strain, a more recent BVDV-2 field isolate, BVDV/TW 2014, was obtained from the Large Animal Hospital, NPUST, Taiwan. The virus strains were propagated in Madin-Darby bovine kidney (MDBK) cells (BCRC 60126; Bioresource Collection and Research Center, Taiwan) using Eagle’s Minimum Essential Medium supplemented with 7% fetal bovine serum. Since the BVDV-2 strains used are non-cytopathic, 50% FAID_50_ was employed to determine virus titer. In 96-well plates, virus sample (100 μL) and MDBK cells (100 μL of 1 × 10^5^/mL) were co-cultured for 6 days at 37 °C, 5% CO_2_. Buffered Formalde-Fresh (Thermo Fisher Scientific, MA, USA) was used for cell fixing and anti-BVDV fluorescein-conjugated polyclonal antiserum (VMRD, WA, USA) at 1:5 dilution was used for virus detection. Fluorescent wells were observed under a fluorescent microscope and BVDV FAID_50_ was calculated using the Reed and Muench method [7].

### Cloning and expression of BVDV E2

A partial segment (nucleotide 55 to 1026 of the full 1116 bps) of the E2 gene was cloned from the BVDV/TW2008 strain using reverse transcription-polymerase chain reaction (RT-PCR), with the following primers: forward: 5′- GCGGGATCCGGGTTATTGGGGCCAGAGAGT-3′, and reverse: 5’-ATAGCGGCCGCTATGAACTCTGAAAAGTAATC-3′. Briefly, TRIzol® Reagent (Thermo Fisher Scientific, MA, USA) was used for viral RNA extraction according to the manufacturer’s instructions. Applied Biosystems High Capacity cDNA Reverse Transcription Kits (Applied Biosystems, CA, USA) were used for cDNA production and PCR reaction was carried out using Ex Taq (Takara, Shiga, Japan) in the Thermocycler (Takara, Shiga, Japan). The E2 PCR products were inserted into the pGM-T vector using the pGM-T Cloning Kit (GeneMark, Taichung, Taiwan) and the sequence of the amplified E2 gene was determined. Using the Clustal W method in MegAlign of the DNAStar software (DNASTAR, WI, USA), the E2 sequence was compared to that of other BVDV strains from Taiwan, China, USA, and Japan.

To produce recombinant baculovirus expressing BVDV E2 protein, the BAC-TO-BAC® Baculovirus Expression System (Invitrogen, CA, USA) was employed following the manufacturer’s instructions. Briefly, using *Bam*HI and *Not*I restriction sites, the E2 gene was subcloned from pGM-T into the pFastBac HT B vector, resulting in pFastBac HT B/E2. pFastBac HT B/E2 was then used to transform DH10Bac competent cells to produce recombinant bacmid DNA, which is the recombinant baculoviral genome. Using the Cellfectin II® Reagent (Invitrogen, CA, USA), the bacmid DNA was then used to transfect Sf9 insect cells for recombinant baculovirus production. The recombinant baculovirus stock was aliquoted in 2% fetal bovine serum (FBS)-Grace’s medium and stored at − 80 °C. For rE2 protein expression, confluent Sf9 cells were infected with recombinant virus stock at multiplicity of infection (MOI) of 0.1 for 5 days at 27 °C and observed for cytopathic effect before cell collection.

For rE2 protein quantitation using sodium dodecyl sulfate-polyacrylamide gel electrophoresis (SDS-PAGE) analysis, 10 μL of protein sample was mixed with 2× sample buffer (100 mM Tri-HCl pH 6.8, 200 mM dithiothreitol, 4% sodium dodecyl sulfate, 0.2% bromophenol, 20% glycerol) in a 1.5 mL Eppendorf tube and boiled for 10 min in hot water. To establish a standard curve, serial dilution of bovine serum albumin (BSA, KPL, MD, USA) was prepared. Gel electrophoresis was performed on the samples and 0.1% Coomassie blue was used for staining for 12 h before decolorization. Image analysis of the resulting gel was performed using Uvitec to obtain a BSA standard curve. Protein concentration was deduced using regression analysis.

To evaluate the antigenicity of rE2 using Western blotting, after gel electrophoresis, protein samples were transferred onto polyvinylidene difluoride (PVDF) membranes (Amersham biosciences, Buckinghamshire, UK) using the TE22 Mini Tank Transfer Unit (GE Healthcare, WI, USA), with transfer buffer (25 mM Tris, 192 mM glycine, 20% methanol) run at 300 mA. After transfer, PVDF membranes were blocked with 1X BSA blocking buffer (KPL/SeraCare, MD, USA) and then washed three times using phosphate buffered saline-Tween 20 (PBST) (1xPBS, 0.5% Tween 20). For the primary antibody, 1:500 dilution of bovine BVDV convalescent serum was used for overnight incubation at 4 °C. After washing three times with PBST, the secondary antibody, 1:5000 dilution of goat anti-bovine IgG-HRP (KPL/SeraCare, MA, USA) was applied for 1 h at 37 °C before washing again with PBST three times. For color development, ECL plus Western Blot Detection Reagents (GE Healthcare, WI, USA) were used according to the manufacturer’s instructions. Results were analyzed using the G Box Chemi-luminescence Imaging System (Syngene, MD, USA).

### Vaccine preparation

Vaccines containing iBVDV and rE2 protein, emulsified in w/o/w adjuvant, were prepared. For rE2 protein preparation, baculovirus in Sf9 cells expressing rE2 proteins were inactivated using 0.3% binary ethylenimine (Sigma-Aldrich, MI, USA) at 37 °C for 10 h. Inactivation reaction was terminated by the addition of 1/10 volume Na_2_S_2_O_3_ (Alfa Aesar, MA, USA). Sterility of the preparation was confirmed by streaking 1 mL of preparation on Tryptone soy agar plates for 14 days at 37 °C to make sure that no growth occurred. Recombinant protein preparation was spun at 1000×g for 10 min and the pellet was collected as the rE2 protein fraction. No protein purification was performed. For iBVDV preparation, 24 h of BEI inactivation was performed and sterility of the sample was also confirmed as above.

For single component vaccines, 1 × 10^8^ FAID_50_/mL iBVDV/TW2014 and 200 μg/mL rE2 protein were prepared. For vaccine containing both iBVDV and rE2, concentrated rE2 preparation was added to iBVDV to achieve the same final concentrations as the single component vaccines. Finally, antigen preparations were mixed with the adjuvant ISA 206 VG (SEPPIC, Puteaux, France) in a 1:1 (*v*/v) ratio using Top Three-way Stopcocks (Meditop, Persiaran Usahawan, Malaysia). The prepared vaccines were stored at 4 °C for animal experiments.

### Immunization and challenge

Twelve 4–5-month-old, BVDV-negative goats (native to Taiwan, raised at NPUST farms), as confirmed using the LSIVet™ BVD/BD p80 Blocking One Step Kit (Thermo Fisher Scientific, MA, USA), were randomly divided into four groups of three animals each and immunized twice, 2 weeks apart, intramuscularly with one of the four vaccine preparations: 1) iBVDV+rE2, 2) rE2, 3) iBVDV, and 4) saline, at 2 mL per dose. On week 4 after primary immunization, the four groups were intranasally challenged with 2 mL of 1 × 10^8^ FAID_50_/mL BVDV/TW2014. For immune response evaluation, blood was collected on week 0, 2, and 4 post primary immunization for antibody and peripheral bone marrow cells (PBMCs) analyses. After challenge, blood samples were collected daily for analyses of leukopenia and viremia. Temperatures and clinical signs were also recorded daily after challenge. Goats were sacrificed 3 weeks after challenge by intravenous overdose of barbiturate. Animal experiments were carried out at the NPUST Barrier and Containment Facility, and all experimental protocols for animal trials were approved by the NPUST Animal Care and Use Committee.

### Antibody analysis

Whole blood was collected and spun at 800×g, 4 °C, for 10 min to separate coagulated blood cells from the serum, which was collected for ELISA analysis. Flat-bottomed 96-well plates were coated with inactivated BVDV/TW2014 (1.25 μg/mL) in the coating buffer (15 mM Na_2_CO_3_, 35 mM NaHCO_3_, 3 mM NaNO_3_, pH = 9.6) at 4 °C overnight. The plates were washed with PBST (0.5% Tween) 3 times and blocked by 1% BSA in PBST at 37 °C for 1 h. After blocking, goat serum samples from the four experimental groups, diluted 1:4000 with PBS, were added into the wells (100 μL/well) and incubated at 37 °C for 1.5 h. Serum from the saline group acted as negative control. Thereafter, the plates were washed 3 times with PBST, and rabbit anti-goat IgG HRP at 1:10,000 dilution (KPL) was added (100 μl/well). The plates were incubated at 37 °C for 1 h, washed 3 times with PBST, and 100 μL TMB 2-component Microwell Peroxidase substrate (KPL) was added to each well. The reaction was stopped by adding 100 μL of TMB stop solution (KPL) after 5 min and the plates were read at 450 nm by a multi-well plate reader.

### T-cell proliferation assay

To collect goat PBMCs for T-cell proliferation assay, whole blood was collected in ethylenediaminetetraacetic acid (EDTA, an anti-coagulant) tubes and spun at 800×g, 4 °C, for 10 min. The upper layer of serum was removed and the remaining blood cells were resuspended in equal volumes of PBS. The resuspended blood cells were then layered slowly on an equal volume Ficoll-Paque™ PLUS (GE Healthcare, Stgiles, Sweden) in a 50 mL tube and spun at 1500×g, 4 °C, for 40 min. The resulting middle PBMC layer was collected, rid of red blood cells using 1% acetic acid, washed with PBS, and resuspended in RPMI-1640 (10% FBS and 0.05 mM ·-mercaptoethanol). For T-cell proliferation assay, in 96-well plates, goat PBMCs (100 μL of 1 × 10^6^ cell/mL) were seeded and stimulated with 10 μg/well of iBVDV for 72 h. Concanavalin A (ConA) (Sigma-Aldrich, MI, USA) at 1 mg/mL was used as positive control and non-stimulated cells acted as negative control. The proliferation extent of T-cells was then measured using the CellTiter 96® AQueous One Solution Cell Proliferation Assay (Promega, WI, USA), per the manufacturer’s instructions. OD_492nm_ was read and stimulation index (SI) calculated, SI = (OD of treatment − OD of background)/(OD of the negative control − OD of background). Wells containing only RPMI-1640 and 20 mL of tetrazolium acted as the background level.

### Clinical evaluation

Blinded clinical examinations were performed daily after BVDV challenge. Severity of clinical signs were judged by a veterinarian and assigned a numerical value according to Table 1 [8]. Daily clinical scores were calculated by summing up the numerical values of the five clinical parameters.

**Table 1:** Parameters used to calculate clinical scores in BVDV-infected goats

Clinical parameter	State description	Numerical value
General state	Normal	0
	Moves slowly, head down	1
	Lying down/staggers	2
	Recumbent	3
Appetite	Normal	0
	Reduced	1
	Absent	2
Rectal temperature	< 39.5 °C	0
	39.5 °C -39.9 °C	1
	≥ 40.0 °C	2
Nasal discharge	Normal	0
	Slight uni−/bilat. Serous	1
	Moderate bilat. Serous to purulent	2
	Copious bilat. Purulent	3
Feces state	Normal	0
	Celiac affection	2
	Mucohemorrhagic diarrhea	4

### Hematology

To determine if leukopenia occurred, 0.5 mL of whole blood was collected daily post-challenge in EDTA tubes and 1% acetic acid was added to lyse red blood cells. White blood cells were counted under the microscope at 200X magnification.

### Virus detection

Viremia was detected using quantitative RT-PCR (RT-qPCR). RNA was extracted from collected whole blood (in EDTA tubes) using the Total RNA Mini Kit (Geneaid Biotech, New Taipei City, Taiwan). Applied Biosystems High Capacity cDNA Reverse Transcription Kits (Applied Biosystems, Foster City, CA, USA) was used for cDNA production. qPCR detection of viral cDNA was performed using a continuous fluorescence detector with the SYBR1 Green Realtime PCR Master Mix (Takara, Shiga, Japan). Primers used are based on the 5′ untranslated region of BVDV, forward 5’-GCCATGCCCTTAGTAGGACTAGC-3′, reverse: 5′- CAACTCCATGTGCCATGTACAGC-3′ [9]. To control for mRNA levels, data were normalized against the values obtained for β-actin.

### Statistical analysis

Statistical analysis was performed using SAS 9.0 general linear models and Duncan’s Multiple Range Test was used for comparison between groups, with different letters indicating statistical significance, *p* < 0.05. Data are shown as mean ± standard deviation. To test for statistically significant difference in viremia for the vaccine groups, viremia results from 7 to 13 days post challenge were compared using the Pearson chi-square test in the software Statistica V.13.

## Results

### iBVDV and rE2 were prepared as vaccines

**Fig. 1:**
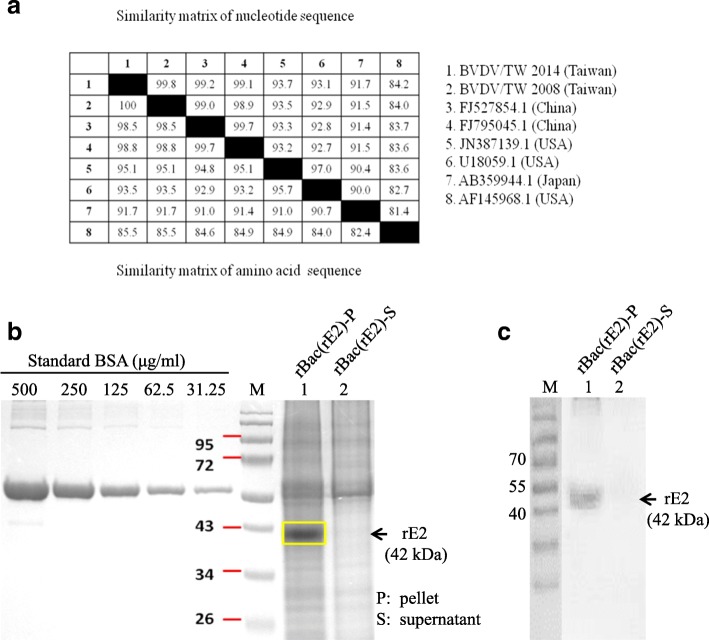
Cloning and expression of the BVDV E2 gene. The E2 gene of BVDV/TW2008 was cloned for expression in a baculovirus expression system and the sequence was compared to that of other BVDV-2 strains (**a**). Expression and quantitation recombinant E2 (rE2) was done using SDS-PAGE analysis (**b**). Antigenicity was verified using bovine convalescent anti-serum (**c**)

The E2 gene, excluding the c-terminal transmembrane domain, of BVDV/TW2008 was cloned for protein expression in a baculovirus expression system. The sequence of the cloned E2 gene was compared to that of other strains (Fig. 1a) to determine if the BVDV/TW2008 E2 gene would still be protective when expressed as an antigen. When compared to the E2 sequence of BVDV/TW2014, which was used as the challenge strain in this study, nucleotide sequences showed 99.8% identity and amino acid sequences showed 100% identity, suggesting that the cloned E2 gene will be protective against the challenge strain. Expressed rE2 moved at the 42 kDa position and quantitation was done using SDS-PAGE analysis (Fig. 1b). Antigenicity of rE2 was verified with convalescent bovine anti-sera (Fig. 1c). Four different formulations were prepared from iBVDV and rE2, with w/o/w adjuvant: 1) iBVDV+rE2, 2) rE2, 3) iBVDV, and 4) saline as negative control.

### iBVDV+rE2 vaccine enhanced cell-mediated immunity

**Fig. 2:**
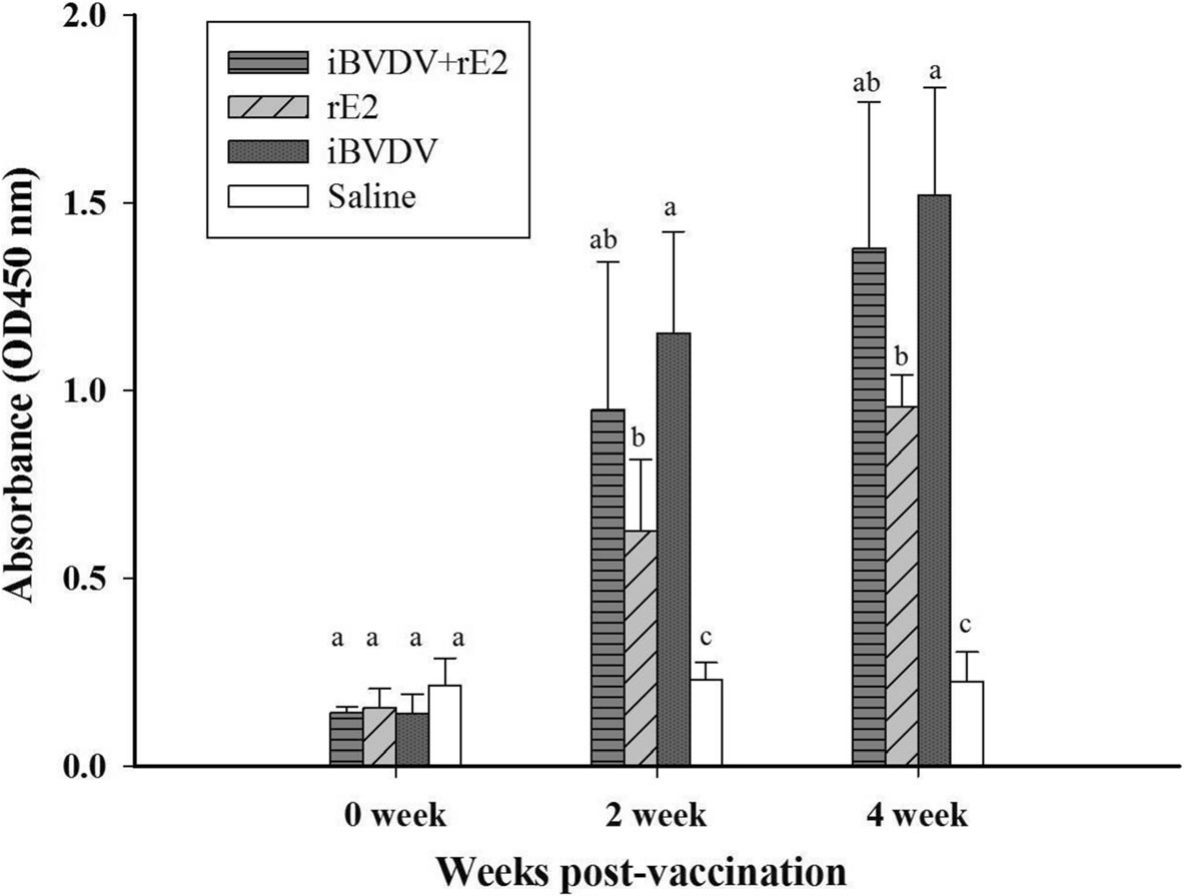
Antibody response in goats immunized with BVDV vaccines. Goats were immunized twice intramuscularly with four different vaccine formulations: 1) iBVDV+rE2, 2) rE2, 3) iBVDV, and 4) saline. Total anti-BVDV IgG levels were analyzed by ELISA. Data represent means ± SD. Differences between groups were analyzed by Duncan’s significance test (Different letters indicate significant difference at *p* < 0.05)

**Fig. 3:**
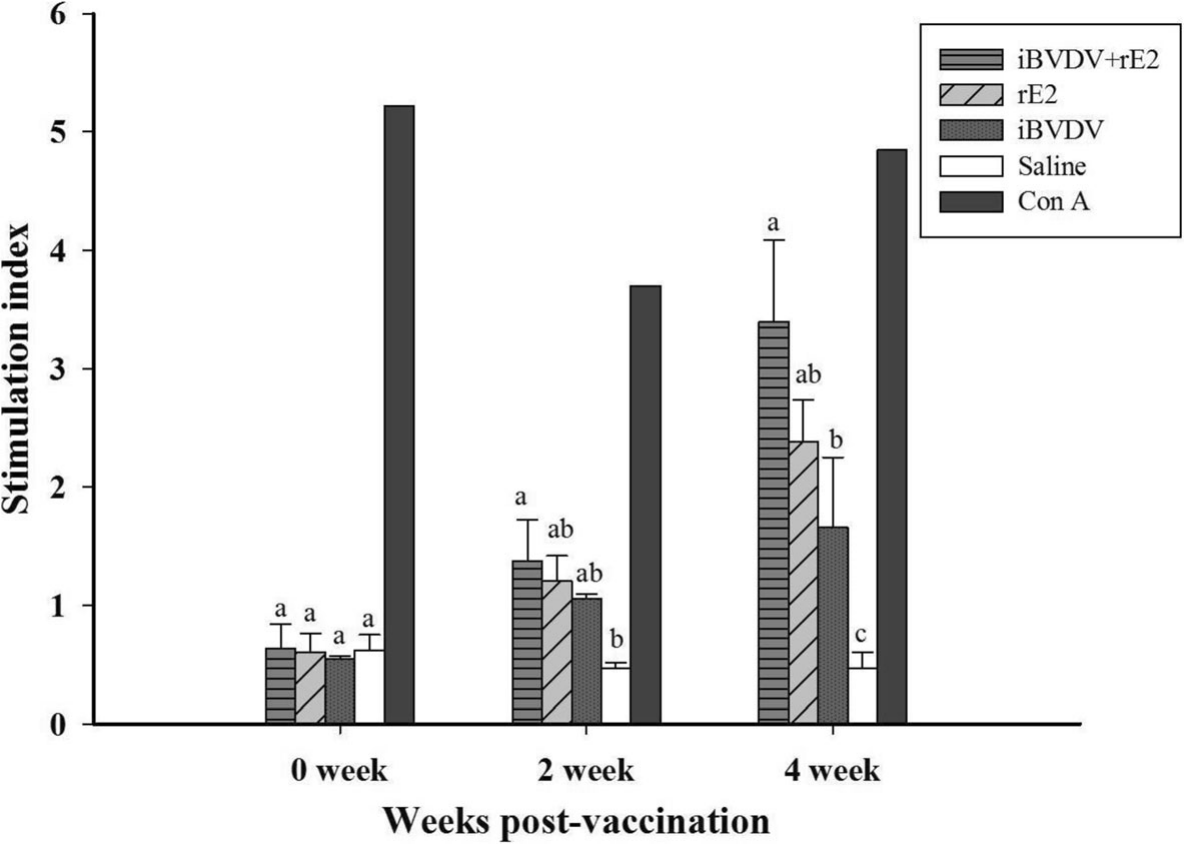
T-cell proliferation assay of PBMC from goats immunized with BVDV vaccines. Goats were immunized twice intramuscularly with four different vaccine formulations: 1) iBVDV+rE2, 2) rE2, 3) iBVDV, and 4) saline. PBMC from blood were isolated and stimulated with inactivated BVDV or Con A (as a positive control). Proliferation extent was measured and stimulation index (SI) calculated. Data represent means ± SD. Differences between groups were analyzed by Duncan’s significance test and different letters indicate significant difference at *p* < 0.05

To evaluate the immune response elicited by the formulated vaccines, four groups of BVDV-naïve goats, three in each group, were immunized and boosted 2 weeks apart with the four different formulations mentioned above. Analysis showed that, compared to that of the saline group, serum anti-BVDV antibody levels increased after vaccination for the vaccine groups (Fig. 2). However, vaccine containing both iBVDV and rE2 did not elicit higher antibody levels than vaccines with iBVDV or rE2 alone. In terms of cellular immunity, T-cell proliferation assay showed that the addition of rE2 enhanced T-cell response elicited by iBVDV 4 weeks post vaccination (Fig. 3).

### iBVDV+rE2 vaccine showed improved protection against BVDV challenge

**Fig. 4:**
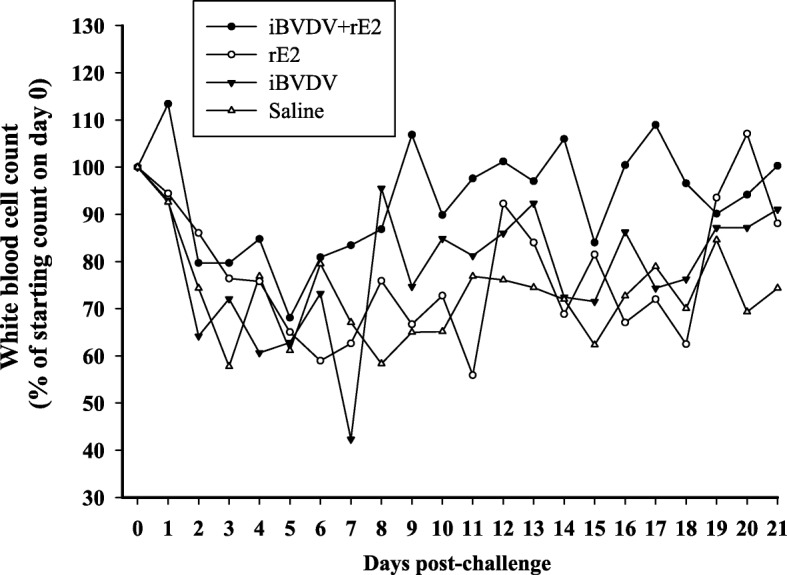
Leukopenia of immunized goats following BVDV challenge. Goats were immunized twice intramuscularly with four different vaccine formulations: 1) iBVDV+rE2, 2) rE2, 3) iBVDV, and 4) saline, and challenged with 1 × 10^8^ FAID_50_ BVDV/TW 2014 4 weeks after primary immunization. Number of white blood cells in blood was counted. Means are presented

**Fig. 5:**
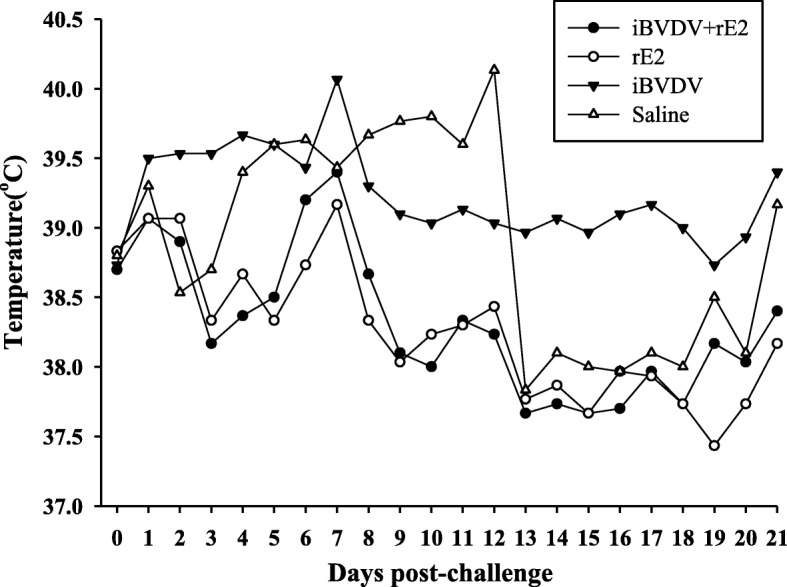
Pyrexia of immunized goats following BVDV challenge. Goats were immunized twice intramuscularly with four different vaccine formulations: 1) iBVDV+rE2, 2) rE2, 3) iBVDV, and 4) saline, and challenged with 1 × 10^8^ FAID_50_ BVDV/TW 2014 4 weeks after primary immunization. Rectal temperatures after challenge were recorded. Means are presented

**Fig. 6:**
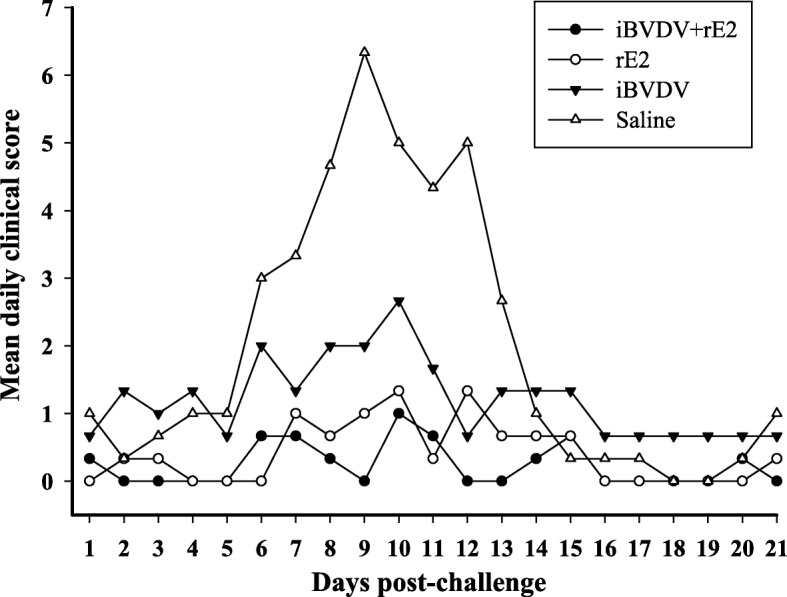
Mean daily clinical scores of immunized goats following BVDV challenge. Goats were immunized twice intramuscularly with four different vaccine formulations: 1) iBVDV+rE2, 2) rE2, 3) iBVDV, and 4) saline, and challenged with 1 × 10^8^ FAID_50_ BVDV/TW 2014 4 weeks after primary immunization. Clinical signs were recorded and mean daily clinical scores calculated

For protective efficacy evaluation, the four immunized groups were challenged with BVDV/TW 2014. In one of the first signs of BVDV infection, leukopenia, a 40% reduction in white blood cell count was observed in the saline group 3 days post challenge (Fig. 4). For goats receiving the iBVDV+rE2 vaccine, white blood cell count dropped by only about 20% initially, and then recovered at a faster pace and to a fuller extent than that of the saline group. In terms of pyrexia, rectal temperatures of goats vaccinated with iBVDV+rE2 remained below 39.5 °C, in contrast to the elevated temperature observed for the saline group between 7 and 14 days after challenge (Fig. 5). Observation of clinical signs showed that the iBVDV+rE2 vaccine achieved the most reduction in clinical scores between 7 and 14 days after challenge (Fig. 6). Overall, for the parameters of leukopenia, pyrexia, and clinical signs, the double-component iBVDV+rE2 vaccine showed similar efficacy as the single-component vaccines.

Differences in viremia were observed, however, between the vaccine groups. Using RT-qPCR to detect BVDV presence in immunized goats after challenge, results showed that the iBVDV+rE2 vaccine eliminated virus presence in all three goats. However, for the iBVDV group, viremia was detectable in two out of three goats 7–13 days after challenge. For the rE2 group, viremia was detectable in one out of three goats (Table 2). We conclude that the addition of rE2 enhances the efficacy of iBVDV vaccines by reducing virus replication.

**Table 2:** Viremia of immunized goats following BVDV challenge using RT-qPCR

Goat No.	^a^Days post-challenge														
	1	3	5	7	8	9	10	11	12	13	14	15	17	19	21
iBVDV+rE2															
241	–	–	–	–	–	–	–	–	–	–	–	–	–	–	–
250	–	–	–	–	–	–	–	–	–	–	–	–	–	–	–
546	–	–	–	–	–	–	–	–	–	–	–	–	–	–	–
rE2															
541	–	–	–	–	–	–	–	–	–	–	–	–	–	–	–
542	–	–	–	+	–	+	–	+	+	+^b^	–	–	–	+	–
543	–	–	–	–	–	–	–	–	–	–	–	–	–	–	–
iBVDV															
37	–	–	–	+	–	–	–	–	–	–	–	–	–	–	–
244	–	–	–	–	–	–	–	–	–	+	–	–	–	–	–
246	–	–	–	–	–	–	–	–	–	–	–	–	–	–	–
Saline															
538	–	–	–	–	+	+	+	+	+	+	–	–	–	–	–
539	–	–	–	+	+	+	+	+	+	+	–	–	–	–	–
533	–	–	–	+	+	+	+	+	+	+	–	–	–	–	–

^a^Goats were immunized twice intramuscularly with four different vaccine formulations: 1) inactivated BVDV+rE2, 2) rE2, 3) inactivated BVDV, and 4) saline, and challenged with 1 × 10^8^ FAID_50_ BVDV/TW 2014 4 weeks after primary immunization. Viremia was detected using RT-qPCR for 21 days after challenge for each animal. Samples that reacted with the RT-qPCR were designated positive (+) and non-reactive samples were designated negative (−)

^b^Using the Pearson chi-square test, for viremia between 7 and 13 days post challenge, the *p* value was 0.017 when comparing between iBVDV+rE2 and rE2, and 0.147 when comparing between iBVDV+rE2 and iBVDV

## Discussion

Our study sought to improve the protective efficacy of iBVDV vaccines by enhancing humoral immunity against the main antigen, E2, and results showed that the addition of rE2 to iBVDV vaccines abrogated virus presence in whole blood. The absence of BVDV viremia bodes well for potential sterilizing immunity. However, antigenic variation of E2 may lead to partial protection in a heterologous challenge. As shown in the E2 sequence comparison in Fig. 1a, up to 17.6% divergence in amino acid sequence can be seen among various strains. As divergence of the E2 sequence increases, effective vaccines might require the inclusion of E2 proteins of divergent strains, or other BVDV proteins.

Since inactivated pathogens and protein antigens are usually presented exogenously to the immune system, it can be expected that inactivated and subunit BVDV vaccines elicited very little cellular immunity. To further improve protective efficacy of these vaccines, the induction of stronger cellular immunity may be considered. Evidence showed that cellular immunity to BVDV can provide protection. In studies done on calves with high levels of maternal antibody, T cell responses were shown to be sufficient for protection in the absence of measureable humoral response [10–12]. Also, modified live BVDV vaccines, deemed more protective than inactivated or subunit vaccines, elicited antigen-specific activation of T lymphocyte subsets while a killed vaccine did not. Therefore, endogenous presentation (through DNA vaccination, for example) of important T cell epitopes within the capsid, nonstructural proteins NS2/3, or E^rns^ may contribute significantly to protection by eliciting cellular immunity against virus-infected cells. DNA vaccination using E2 [13] and NS3 [14] sequences was shown to induce protective T cell responses. By increasing cellular immunity, the range and duration of inactivated and subunit vaccines may be improved.

When studying BVDV biology, researchers have looked for alternative animal models since experimenting on pregnant cattle is cost-prohibitive. Because BVDV infection can result in abortions, it is important to study congenital BVDV-2 infection, which in turn necessitates more economical animal models. A study has demonstrated that the biology of BVDV-2 infection is essentially similar in pregnant sheep and cattle [15]. For our study, challenge experiments were performed in goats since natural infection of BVDV in goats has been reported [16, 17] and experimental infection has also been performed previously, causing reproductive diseases [18]. Because the course of disease of BVDV is essentially similar in cattle and goats, vaccine protective efficacy results obtained from a goat model offers reference value. Following our current study, vaccine efficacy studies can also be performed on pregnant goats.

## Conclusions

Our study showed that the addition of rE2 protein may be an effective way to enhance the protective efficacy of inactivated BVDV vaccines, and this improved vaccine could prove useful in a BVDV control program.

## Abbreviations

BSA: Bovine serum albumin
BVDV: Bovine viral diarrhea virus
EDTA: Ethylenediaminetetraacetic acid
FAID_50_: 50% fluorescent antibody infectious dose
FBS: Fetal bovine serum
iBVDV: Inactivated bovine viral diarrhea virus
MDBK: Madin-Darby bovine kidney
MOI: Multiplicity of infection
PBMC: Peripheral bone marrow cells
PBST: Phosphate buffered saline-Tween 20
PVDF: Polyvinylidene difluoride
rE2: Recombinant E2
RT-PCR: Reverse transcription-polymerase chain reaction
RT-qPCR: Reverse transcription-quantitative polymerase chain reaction
SDS-PAGE: Sodium dodecyl sulfate-polyacrylamide gel electrophoresis
w/o/w: Water-in-oil-in-water
